# The age-related decline in spatiotemporal gait characteristics is moderated by concerns of falling, history of falls & diseases, and sociodemographic-anthropometric characteristics in 60–94 years old adults

**DOI:** 10.1186/s11556-021-00275-9

**Published:** 2021-10-05

**Authors:** Daniel Niederer, Tobias Engeroff, Johannes Fleckenstein, Oliver Vogel, Lutz Vogt

**Affiliations:** 1 Department of Sports Medicine and Exercise Physiology, Goethe University Frankfurt am Main, Frankfurt am Main, Germany; 2 Division Health and Performance, Goethe University Frankfurt, Institute of Occupational, Social and Environmental Medicine, Frankfurt, Germany; 3 Institute of Human Movement Science, University of Hamburg, Hamburg, Germany

**Keywords:** Fear of falling, Older adults, Fall risk, Walking interaction

## Abstract

**Background:**

Associations between age, concerns or history of falling, and various gait parameters are evident. Limited research, however, exists on how such variables moderate the age-related decline in gait characteristics. The purpose of the present study was to investigate the moderating effects of concerns of falling (formerly referred to as fear of falling), history of falls & diseases, and sociodemographic characteristics on changes in gait characteristics with increasing age in the elderly.

**Methods:**

In this individual participant level data re-analysis, data from 198 participants (*n* = 125 females) from 60 to 94 years of age were analysed (mean 73.9, standard deviation 7.7 years). Dependent variables were major spatiotemporal gait characteristics, assessed using a capacitive force measurement platform (zebris FDM-T). Age (independent variable) and the moderating variables concerns of falling (FES-I), gender/sex, history of falls and fall-related medical records, number of drugs daily taken, and body mass index were used in the statistical analysis. Hierarchical linear mixed moderation models (multilevel analysis) with stepwise (forward) modelling were performed.

**Results:**

Decreases of gait speed (estimate = −.03, equals a decrease of 0.03 m/s per year of ageing), absolute (− 1.4) and gait speed-normalized (−.52) stride length, step width (−.08), as well as increases in speed normalized cadence (.65) and gait speed variability (.15) are all age-related (each *p* < .05). Overall and specific situation-related concerns of falling (estimates: −.0012 to −.07) were significant moderators. History of potentially gait- and/or falls-affecting diseases accelerated the age-related decline in gait speed (−.002) and its variability (.03). History of falls was, although non-significant, a relevant moderator (in view of increasing the model fit) for cadence (.058) and gait speed (−.0027). Sociodemographics and anthropometrics showed further moderating effects (sex moderated the ageing effect on stride length, .08; height moderated the effect on the normalised stride length, .26; BMI moderated the effects on step width, .003). .

**Conclusion:**

Age-related decline in spatiotemporal gait characteristics is moderated by concerns of falling, (non-significantly) by history of falls, significantly by history of diseases, and sociodemographic characteristics in 60–94 years old adults. Knowing the interactive contributions to gait impairments could be helpful for tailoring interventions for the prevention of falls.

**Trial registration:**

Re-analysis of [21–24].

## Introduction

The risk for falls increases with increasing age, in particular above the age of 65 years [1, 2]. One third of all adults aged over 65 fall at least once a year [1]. In this population, falls cause 20–30% of mild-to-severe injuries. More than half of the fallers who need treatment require hospitalization [3]. Consequently, the “prevention of falls among the elderly is arguably one of the most important public health issues in today’s aging society” [4].

To prevent falls, modifiable causes and risk factors for a fall, as well as their interaction and pathways must be known and targeted in effective strategies [5]. Beyond non-modifiable risk factors, such as sex/gender [6] and increasing age [2], numerous modifiable or at least partially modifiable factors are known. From a disease-viewpoint, cognitive impairments and dementia [7], sarcopenia [8] or a history of potentially fall-affecting disease(s) [9] are reported factors. Furthermore, adverse effects from medication like psychotropics [10], loop diuretics [11], opioids and antiepileptics and the detrimental effect of multi-medication [12] are risk factors. In the inter-factorial continuum of falls, psychological issues like concerns of falling, a recent history of falling, and rather biological limitations of physical function or mobility are known to be contributors for falls [2, 13].

These different risk factors must be treated interactively and not as isolated contributors. Mobility and physical function impairments, often operationalised by spatiotemporal gait parameters [14], for example, are strongly associated with concerns of falling. More detailed, impairments in spatiotemporal characteristics such as gait speed and stride length, as well as impairments in stride width or double limb support time [15, 16] are known regressors of concerns of falling. Ageing itself leads to adaptations in gait. Impairments in spatiotemporal characteristics are, when they are larger than default age-related declines in spatiotemporal gait parameters, predictive for a fall [15, 17]. Changes in these gait characteristics, which are beyond age-related changes, are, on the one hand, likely to be an individual’s adaptation in stability and posture following concerns of falling. On the other hand, such changes are also potentially associated to a history of falls [18, 19]: Age, frailty, history of falls, sex, and polypharmacy are related to gait parameters. The fall itself is likely to be triggered by functional impairment, in particular during walking [20].

Conclusively, ageing leads to age-related, less than age-related, or beyond age-related declines in (inter alia) gait characteristics. This association may be moderated by numerous risk factors for falls. The interactive association of ageing, falls-related functional changes and biopsychosocial risk factors for falls has, however, not yet been finally delineated. The purpose of the present analysis was to provide further evidence on this complex interactive structure. In line with the above-mentioned rationale, analyses on the temporal structure (ageing is followed by gait changes) and the most plausible interaction structure is missing so far. In this study, we investigated the moderating effects of concerns of falling, history of falls & diseases, and sociodemographic characteristics on gait changes with increasing age in the elderly. We hypothesise that age-related declines in spatiotemporal gait characteristics are moderated by numerous fall risk factors like concerns of falling, polypharmacy, a history of falls, and having a history of potentially fall-related diseases.

## Methods

### Study design and ethics

In this cross-sectional retrospective trial, all data included were obtained from previously published research or study samples included in the publishing process (individual participant level data re-analysis). Individual participant data were obtained from three multicentre randomized trials [21–24] and re-analysed. Gait data was not the focus of these previous publications. One of the studies published data regarding gait speed [21]. The other two studies sampled gait characteristics as secondary outcomes but did not include this data in a manuscript so far (study 2: [22, 24]), or remains unpublished [23] (as of July 2021).

The analysis was conducted using anonymized individual participants’ data. All underlying studies have been approved by an ethical review committee for research (Local ethics committee of the Department 05 of the Goethe University of Frankfurt am Main: 2011–12, Medical ethics committee of the Goethe University of Frankfurt am Main, Germany: 107/13, ethics committee of the Hamburg Chamber of Physicians: PV5762). They, further,were conducted in accordance to the ethical standards set by the declaration of Helsinki (Helsinki, 1964; Fortaleza, 2013). The possibility to re-analyse the assessed but anonymised data was provided by the institutional review boards’ positive hearing.

### Participants

Inclusion criteria were consistent in the three samples: age 60 years and above, being capable of walking, and living in a community dwelling, assisted living facility, or nursing residence housing situation. Exclusion criteria consists of uncorrected visus impairments, dementia, acute injuries or infections, or further severe diseases like unstable angina pectoris, vascular disease of the extremities, or severe cardiopulmonary dysfunction. More details on the exclusion criteria have been described elsewhere [22]. Following the application of inclusion and exclusion criteria, each participant signed informed consent prior to study enrolment. Informed consent included the approval for the re-analysis of the (after study completion) anonymised variables.

Recruitment was conducted by personal contact, by flyers and newspaper bulletins. The participants were recruited in senior residence institutions [22–24] and in two communities [21](95 participants) in the greater Frankfurt metropolitan region (Hessen, Germany). Community dwelling guests ot the residences were, partially, also included [23] Overall, 198 adults (males = 73, females = 125) from 60 to 94 years (mean 73.9, standard deviation 7.7 years; independent variable) were included.

### Outcomes

#### Model definition and temporal structure

The independent variable was age [years]. Moderators were sociodemographic & anthropometric data (sex/gender, body height & weight, body mass index), concerns of falling (sum score and during specific situations), history of fall-related diseases, multi-medication/polypharmacy, and history of fall(s). The dependent variables (different models) were the spatiotemporal gait characteristics variables.

Age and moderating sociodemographic characteristics were asked first, followed by the questionnaires (also moderators); the assessment was completed assessing the gait characteristics in conclusion.

### Moderators

#### Sociodemographic and anthropometric data were asked or assessed using standard equipment

The history of potentially gait-affecting disease (such as hypertonia, musculoskeletal pain, uncorrected impaired visus, neuropathy, recent cancer) and medication intake were assessed by means of a structured interview. Diseases were afterwards simply dichotomised as: yes = participant has a history of potentially gait-affecting disease versus no = no history of such disease(s) reported. The same was done for the multi-medication: yes = more than three different drugs versus no = intake of three or less different drugs [25].

History of falls in the last 6 months was assessed with a self-report questionnaire by means of a structured interview. Sufficient validity of this retrospective procedure against a prospective calendar-reported method is given [26]. For the present analysis, the falls in the previous six months were selected and, again, dichotomised as yes = at least one fall in the previous six month versus no = no such event in this timespan.

Activity-related concerns of falling were captured by means of the German version of the Falls Efficacy Scale (Short FES-I). The FES-I assesses one’s confidence (1 = not concerned, 4 = very concerned) in performing different basic or more demanding physical and social activities of daily living without falling. A sum score is built by simply sum these numbers up. The 7-item short version [23] or the 16-item full version [21, 22, 24] of the FES-I was used. The latter was applied both in complete form and reduced from 16 to the congruent 7 items. The 7 items (activities) are concern of falling during: getting dressed or undressed – taking a bath or a shower – getting in or out of a chair – going up or down stairs – reaching for something above your head or on the ground - walking up or down a slope – going out to a social event. The full 16-item version also assesses concerns during Cleaning the house - Preparing simple meals - Going to the shop - Walking around outside - Answering the telephone - Walking on a slippery surface - Visiting a friend/relative - Going to a place with crowds - Walking on an uneven surface [27]. Psychometric properties of the full and short version are at least sufficient [27–29]. All questionnaires were completed by assessors in form of interviews.

#### Dependent variable gait characteristics

The same setting and device were used for all gait data assessed. A 10-m ground level distance walkway was used. In between the two 4-m acceleration/deceleration walkways, a capacitive force measurement platform of 2-m length was placed (zebris FDM-T, Zebris medical GmbH, Isny, Germany).

The participants walked over the walkway in their habitual/comfortable walking speed. A familiarization trial was followed by 2 [23] to 5 [21] test trials. A valid test trial was defined as containing a minimum of three full footprints (double step). Data was collected with a sampling rate of 50 Hz. The manufacturer’s software (zebris FDM Software Version 1.16.x, Zebris Medical GmbH, Isny, Germany) was used to assess and calculate the following parameters (distance/time between initial heel strikes/contact if not stated other): stride length [cm], gait speed normalized stride length (√v-dependency), step cadence [steps/min], gait speed [m/s], gait speed variability [coefficient of variation, CV, mean/SD], step width [cm] (distance between right and left heel centre), and double stance time [seconds]. Mean values were calculated as outcomes.

### Statistical analyses

All data were displayed descriptively. Interval and pseudo interval scaled data are displayed as means and standard deviations, nominal scaled data as numbers and percentage distributions, ordinal scaled data as percentage distributions.

Linear mixed moderation models (multilevel analysis) investigated the impact of the independent variable age on the dependent variables (gait characteristics). Concerns of falling, history of falls & diseases (including multi-medication), and participants characteristics (sex, height, weight, body mass index) were considered as potential mediators of the association of age and gait characteristics. A stepwise (forward) modelling was performed. During the modelling, all potentially relevant moderating variables (sociodemographic & anthropometric data (sex/gender, body height & weight, body mass index), concerns of falling (sum score and during specific situations), history of fall-related diseases, multi-medication/polypharmacy, and history of fall(s)) were prospectively modelled to find the model with the best fit. Variables were excluded if they were not significant within the total model or if they were only non-relevant contributors to the model fit (as displayed by the within-model by the 2-restricted Log Likelihood). Only the final models (those with the best model fit) are displayed. The linear mixed model’s estimates displays the slope (strength of the relationship per respective unit of mass) of the association and can be interpreted like classic regression coefficients.

All analyses were performed using SPSS (Version 24, IBM SPSS, USA). For all inference statistical analyses, an alpha-error of 5% was considered as a valid cut-off for significance testing. *P*-values below 5% are defined as statistically significant.

## Results

Overall, 198 participants were included. Sixty-three thereof live in nursing homes, the other 135 were community-dwelling or independently-living. The age (independent variable) ranged between 60 and 94 years. Detailed sociodemographic and anthropometric characteristics (moderators) of the participants are given in Table 1. Additionally, Table 1 displays data on concerns of falling, history of falls & diseases (moderators), as well as all spatiotemporal gait characteristics (dependent variables).

**Table 1:** Overview of all data included into the present study. Sociodemographic, concerns of falling, history of falls & diseases, as well as spatiotemporal gait characteristics of the participants are displayed. Interval and pseudo interval data are displayed as means and standard deviations, nominal scaled data as numbers and percentage distribution, ordinal scaled data as percentage distributions

- A – Interval scaled data				
Outcome	Unit	Mean	Standard deviation	Statistical Function
Age	[years]	73.9	7.7	Independent variable
Height	[cm]	164	22	Sample descriptor
Body weight	[kg]	72.8	14.2	Sample descriptor
Body mass index	[kg/m^2^]	26.1	4.3	Moderator
FES-I-7 Concerns of falling sum score*	[points]	8.6	2.1	Moderator
FES-I-16 Concerns of falling sum score*^,^ **	[points]	19.4	3.5	Moderator
Step width	[cm]	10.5	3.4	Dependent Variable
Stride length	[cm]	114	17	Dependent Variable
Stride length – gait speed normalized	[cm]	59.5	5.3	Dependent Variable
Double stance time	[% of total gait cycle]	29	5	Dependent Variable
Step cadence – gait speed normalized	[steps per minute]	55.1	9.0	Dependent Variable
Gait speed	[m/s]	3.7	.83	Dependent Variable
Gait speed Variability Coefficient of Variation	[%]	2.85	3.0	Dependent Variable
- B – Nominal scaled data				
Outcome	Unit	Number / Percentage distribution		Statistical Function
Sex/Gender	[n] / [%]	Female: 125 / 63% Male: 72 /37% Non-binary: 0 / 0%		Moderator
History of fall-related diseases	[n] / [%]	Yes: 50 / 25% No: 148 /75%		Moderator
Multi-Medication	[n] / [%]	Yes: 69 / 35% No: 128 / 65%		Moderator
History of fall(s)	[n] / [%]	Yes: 29 / 14% No: 169 / 86%		Moderator
- C – Ordinal scaled data: relevant Moderators				
Detailed single items of the FES-I: Concerns of falling -	not at all concerned [%]	somewhat concerned [%]	fairly concerned [%]	very concerned [%]
Cleaning **	91	7	1	1
Dressing	82	15	2	1
Meal preparation **	97	3	0	0
Bath or shower	82	5	3	0
Shopping **	95	4	1	0
In or out a Stair	94	4	2	0
Stairs	69	24	7	1
Flat **	97	3	0	0
Reaching	76	20	4	0
Phone call **	91	9	0	0
Slippery walking **	22	57	18	3
Visit **	97	3	0	0
Crowd **	84	14	2	0
Uneven ground **	56	38	5	1
Slope	67	25	7	1
Event	94	4	1	1

n = numbers; * = pseudo-interval-scaled; ** = *n* = 154 (FES-I 16 item only); FES-I-7 = falls efficacy scale 7-item international version; FES-I-16 = falls efficacy scale 16 item international version

**Fig. 1:**
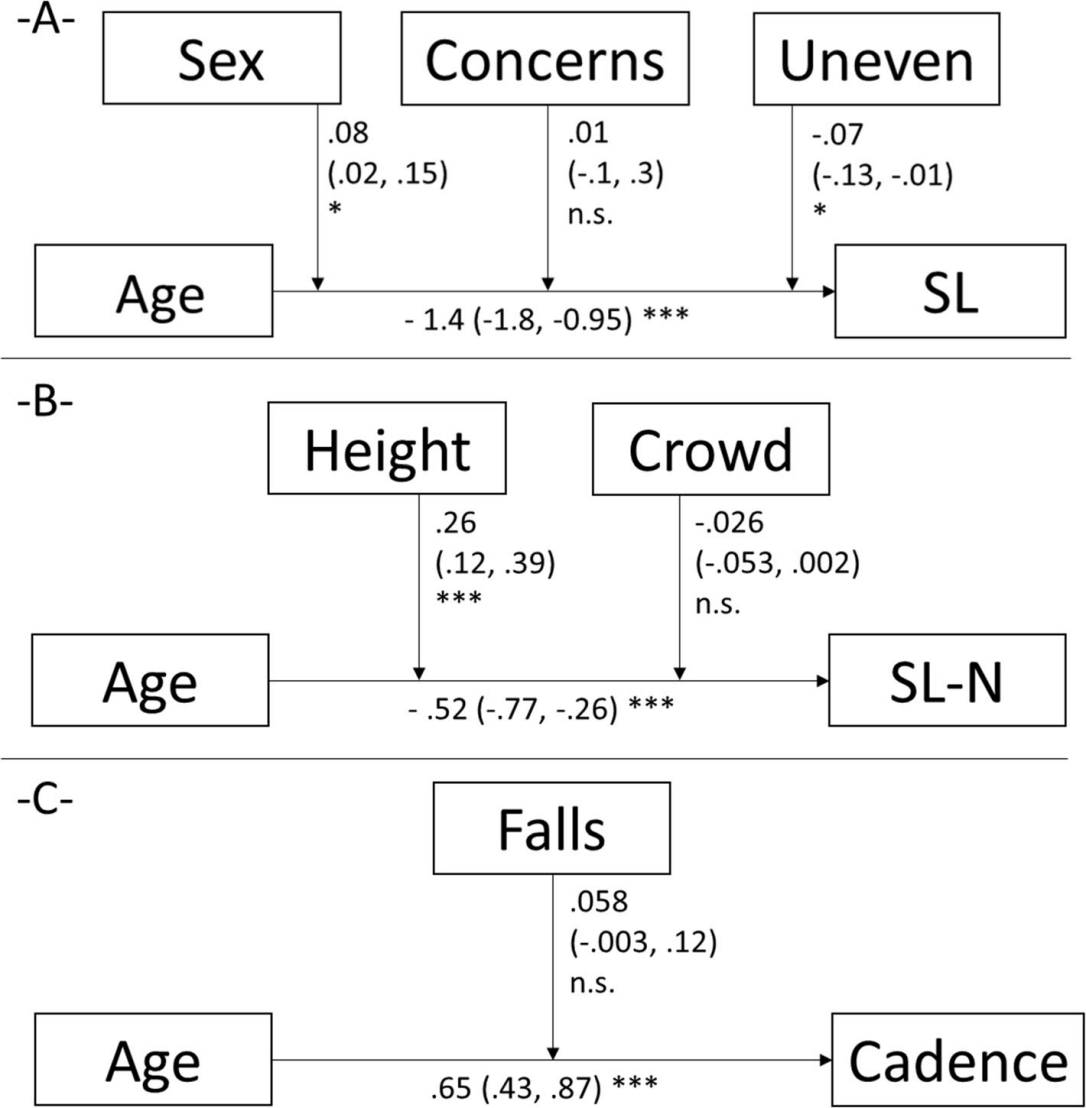
Results of the linear mixed moderation analyses for the dependent variables stride length (-**A**-), gait speed normalized stride length (-**B**-) and step cadence (−-**C**-). In each part, the model with the best fit is displayed (without the excluded variables). Each time, the estimates of how strong the independent variable predicts the dependent variable and of how strong the moderators impacts on this association, are displayed. The estimates (95% confidence intervals), and significance levels are displayed. *, *p* < .05; **, *p* < .01; ***, *p* < .001; SL, stride length; SL-N, gait speed normalized stride length; Concerns, concerns of falling estimated by the FES-I-7 items; Uneven, concerns of falls during walking on uneven surfaces; crowd, concerns of falls during going to a place with crowds

**Fig. 2:**
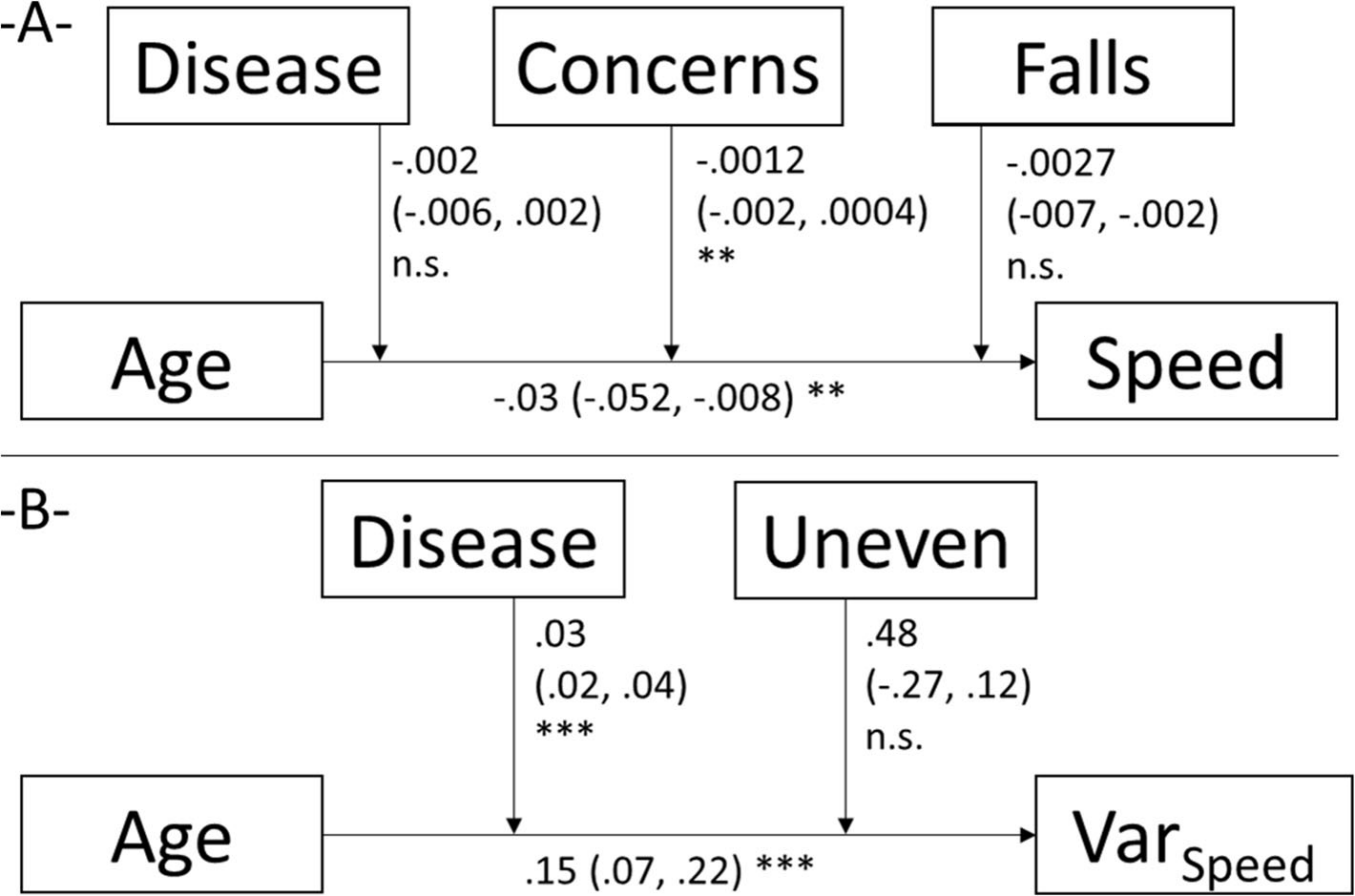
Results of the linear mixed moderation analyses for the dependent variables Gait speed (speed) (**-A**-) and gait speed variability (Var_speed_) (**-B**-). In each part, the model with the best fit is displayed (without the excluded variables). Each time, the estimates of how strong the independent variable predicts the dependent variable and of how strong the moderators impacts on this association, are displayed. The estimates (95% confidence intervals), and significance levels are displayed. *, *p* < .05; **, *p* < .01; ***, *p* < .001; Disease, history of a potentially fall affecting disease; falls, history of fall(s) in the past 6 months; Concerns, concerns of falling estimated by the FES-I-7 items; Uneven, concerns of falls during walking on uneven surfaces

**Fig. 3:**
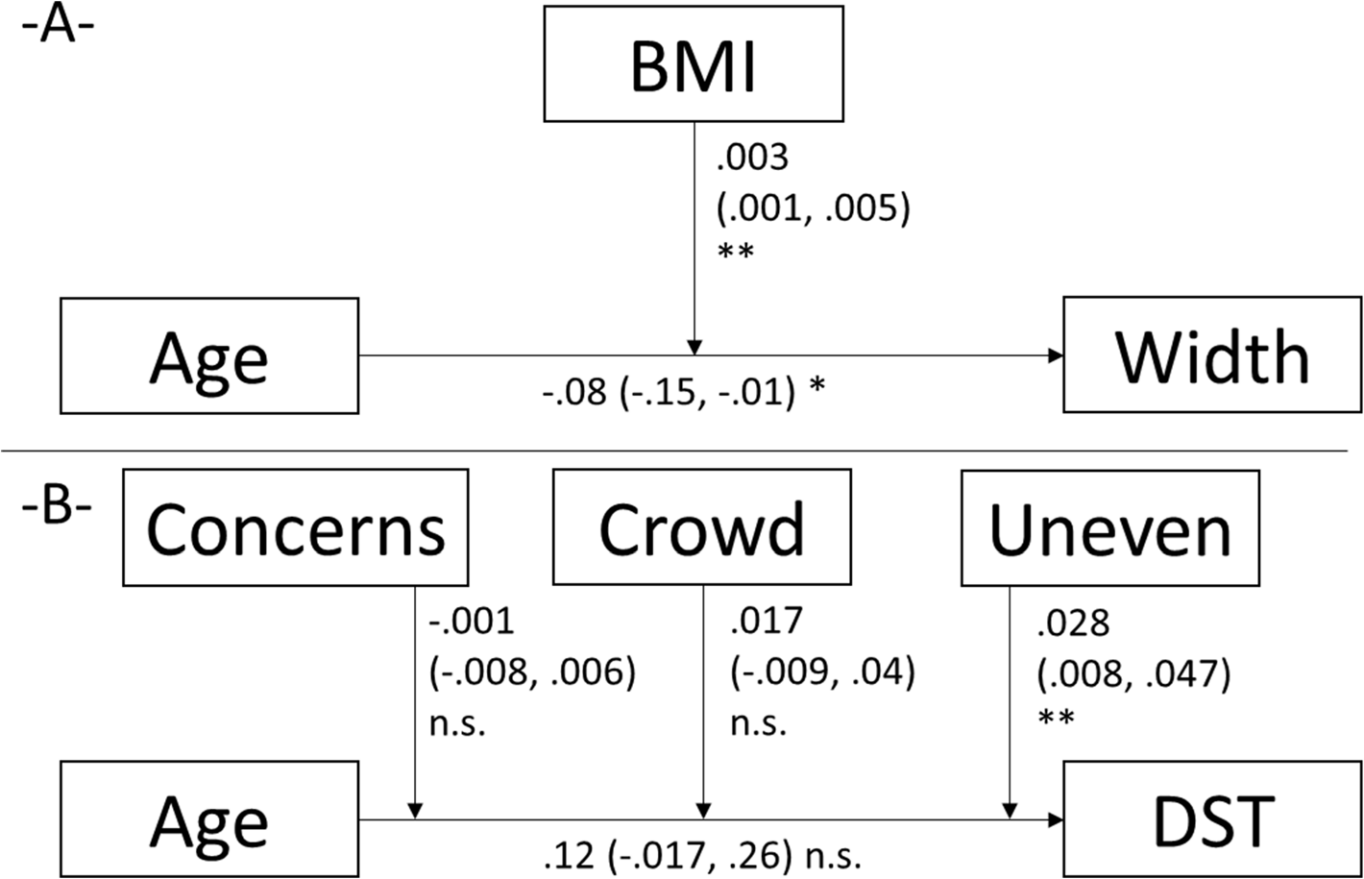
Linear mixed moderation results for the dependent variables step width (width) (-**A**-) and double stance time (-**B**-). In each part, the model with the best fit is displayed (without the excluded variables). Each time, the estimates of how strong the independent variable predicts the dependent variable and of how strong the moderators impacts on this association, are displayed. The estimates (95% confidence intervals), and significance levels are displayed. *, *p* < .05; **, *p* < .01; ***, *p* < .001; BMI, body mass index; Concerns, concerns of falling estimated by the FES-I-7 items; Uneven, concerns of falls during walking on uneven surfaces; Crowd, concerns of falls during walking in a crowd; DST, double stance time

The Figs. 1, 2, 3 display the results of the linear mixed moderation analyses. Decreases of the stride length, absolute and gait speed-normalized, decreases in gait cadence and speed, and increases in gait speed variability were all age-related. These associations were negatively moderated by concerns of falling in five of the seven outcomes. More detailed, general concerns (7-items sum score from the 7- or 16-item FES-I) and specific situation-related (one of the single items) moderated the age-gait association with stride length (sum score), normalized stride length (crowd), gait speed (sum score), gait speed variability (uneven), and double stance time (sum score, crowd, and uneven), Figs. 1, 2, 3). History of falls showed only non-significant contributions on the association of age and gait cadence/speed, a history of potentially gait and/or falls affecting diseases accelerated the age-related decline in gait speed (non-significantly) and the variability in gait speed (positively). Sociodemographic characteristics showed at least one contribution on the age-gait characteristics-association in three models (sex affected the relation of age and stride length, height the impact of age on the stride length and BMI the relation of age and step width).

## Discussion

### Hypothesis verification

Age-related declines in spatiotemporal gait characteristics were moderated by numerous fall risk factors. Single and sum scores of concerns of falling, having a history of potentially fall-related diseases (all significantly) and (non-significantly) a history of falls moderated one or more gait characteristics in 60–94 years old adults. As some of these factors were found to be additional moderators on the decline of gait characteristics with increasing age, the effects are always depend on which gait characteristics is referred to. Our hypothesis could thus be partially verified.

### Contribution to the current evidence

Considering the simple direct effects of aging, decreases of the absolute and gait speed-normalized stride length and gait speed, as well as increases in cadence and gait speed variability were all age-related. Ageing leads to declines in sensory systems and to a reduced ability to adapt to changes in the environment and therefore limits the ability to maintain balance [30]. Gait, in particular spatiotemporal gait characteristics, is a multidimensional construct including energy supply, movement control and demands on multiple organ systems [31]. Impairments in gait, for example a decreased gait speed, thus are likely to not only reflect deficits in motor function but should be seen as an indicator of impaired vitality itself [31].

With focus on the multilevel moderation, we found that the associations of increasing age and impairments in gait characteristics were partially (negatively) moderated by overall and specific situation-related concerns of falling. A history of potentially gait- or falls affecting diseases further accelerated the age-related decline in gait characteristics such as gait speed and gait speed variability. In contrast, history of falls showed only non-significant contributions on the association of age and gait cadence and gait speed. Our data underlines that the different risk factors found in the present study and known in the literature must be rated interactively and not as isolated contributors. The association of concerns of falling we found, for example, confirms the literature on findings of fall concern-associated gait impairments like decreased gait speed, stride length (sum score only non-significantly in our findings), and double limb support time (sum score only non-significantly in our findings) [15, 16]. In contrast to our findings, beyond-age-related impairments in gait characteristics are likely to be associated to a history of falls [18, 19]. More detailed, fallers were found to walk slower with shorter steps and more variable step times than non-fallers and showed a longer stance phase [32]. As we could not reproduce the latter finding, it remains unknown if the relation between a history of falls and gait characteristics is a surrogate of ageing (or even a surrogate of concerns of falls) or an independent risk factor for falls. The fall itself is likely to be triggered by functional impairment, in particular during walking [20].

Our results suggest a temporal relation beyond simple age-gait-associations for certain gait characteristics and moderators. It seems likely that both, the pathway of how ageing leads to declines in gait characteristics and the magnitude of these declines are moderated by at least one of the moderators investigated (concerns of falling, multi-medication, a history of falls, and having a history of potentially fall-related diseases). Gait assessments and in particular gait variability outcomes are sensitive to identify neural changes [33]. Increased, and thus impaired, gait variability poses a particular challenge for older adults and is linked to an increased risk for falls [30]. These two facts must be considered as hints towards a relevant causal interrelation.

### Practical relevance and implications

Modifiable risk factors for falls and their interaction and pathways must be targeted to prevent a fall [5]. To address modifiable factors, the contributing effect of non-modifiable factors must be known. This approach allows estimating real contribution of modifiable factors. Major non-modifiable factors like increasing age (present study) and [2] could thus be separated from the numerous modifiable or at least partially modifiable factors. From the perspective of disease, cognitive impairments and dementia [7], sarcopenia [8], adverse effects from medication like psychotropics [10], loop diuretics [11], opioid and antiepileptic use and multimedication itself [12] might be better treated when the interaction with aging is known.

Mobility and physical function impairments are often operationalised by spatiotemporal gait parameters [14]. Unimodal treatments against such mobility and physical function impairments were described effective in decreasing the numbers of falls. Exercise therapies [34] and vitamin-D-supplementation [35] are two evidence –based approaches to decrease the risk of falling. A risk reduction of approximately 5% was found in pooling analyses on these approaches. Although relevant, this considerable small effect of unimodal treatments calls for a more holistic approach. One may speculate that targeting individual deficits (from a physical point of view) in gait characteristics or moderators of beyond-age-related gait changes could be helpful. Indeed, meta-analytic evidence showed that reactive and volitional stepping interventions reduce falls among older adults by approximately 50% [36]. As a mechanism explanation, this reduction was caused by improvements in reaction time, gait, balance and balance recovery but not in strength [36]. Comparable effects on (balance and) spatiotemporal gait characteristics were reached by virtual reality training [30].

Sociodemographic and anthropometric characteristics showed minor contributions (sex affected the relation of age and stride length, height the impact of age on the normalized stride length, and BMI the relation of age and step width). The impact of height on the age-gait-relation is somewhat new as a finding. The relevance of sex [6], BMI (i.e. obesity [37]) in fall mechanics, fall prevention, or risk of fall determination are confirmed by further literature. However, and although it is of importance to know these associations and their contributing value to the model, these factors are, mostly (except, for example, the BMI) not modifiable and thus likely to be of minor interest in fall-preventive interventions. If the BMI is not only moderating the age-related gait changes but, when modified, also leads to a decrease in the fall risk, decreasing the BMI could be beneficial in fall-preventing intervention strategies, likewise.

### Weaknesses of the study

Concerns for a fall was assessed using two different versions of the same questionnaire (FES-I). The construct itself and most of the items remains the same for the two types. However, that and the retrospectively collected falls need to be considered as limitations of the present study. As we have not investigated fall risk prospectively, a transfer of our results to fall risk must be considered as theoretical.

Gait and falls are both complex matter and have many contributors. Some were assessed as independent or moderating variables in the present study, some (to control for interindividual variability) were exclusion criteria (such as cognitive decline, pathological muscle atrophy, uncorrected visual impairments). Assessing potential further contributors to falls and gait characteristics such as sensory function, alcohol abuse, orthostasis, hip and knee osteoarthritis [38] would have been helpful to show an even more complete picture.

## Conclusions

Age-related declines in spatiotemporal gait characteristics are moderated by numerous factors in 60–94 years old adults. Concerns of falling, history of falls & diseases, and anthropometric-sociodemographic characteristics were found to be moderators to one or more of the associations between age and gait characteristics. Decreases of absolute and gait speed-normalized stride length, gait speed and step width, as well as increases in speed normalized cadence and gait speed variability are all age-related. Overall or specific situation-related concerns of falling were significant moderators of stride length, speed, variability of gait speed, and double stance time. History of potentially gait- or fall- affecting diseases accelerated the age-related decline in gait speed and its variability. History of falls (gait speed and cadence) was a non-significant moderator. Sociodemographic-anthropometric characteristics were further significant (sex on stride length, height on normalised stride length, BMI on step width) moderators. The knowledge of these interactions may be helpful in unravelling the complexity of the interaction of gait, ageing, and risk factors for falls. Knowing the interactive contributions to gait impairments could be helpful for tailoring future interventions addressing falls.

## Data Availability

The datasets used and/or analysed during the current study available from the corresponding author on reasonable request.

## References

[1] Ageing and Life Course, Weltgesundheitsorganisation. WHO global report on falls prevention in older age. Geneva: World Health Organization Ageing and Life Course Family and Community Health; 2008.

[2] Guirguis-Blake JM, Michael YL, Perdue LA, Coppola EL, Beil TL (2018). Interventions to prevent falls in older adults: updated evidence report and systematic review for the US preventive services task force. JAMA..

[3] Scuffham P, Chaplin S, Legood R (2003). Incidence and costs of unintentional falls in older people in the United Kingdom. J Epidemiol Community Health.

[4] Park S-H (2018). Tools for assessing fall risk in the elderly: a systematic review and meta-analysis. Aging Clin Exp Res.

[5] Walls HL, Peeters A, Reid CM, Liew D, McNeil JJ (2008). Predicting the effectiveness of prevention: a role for epidemiological modeling. J Prim Prev.

[6] El-Menyar A, Tilley E, Al-Thani H, Latifi R (2019). Females fall more from heights but males survive less among a geriatric population: insights from an American level 1 trauma center. BMC Geriatr.

[7] Fernando E, Fraser M, Hendriksen J, Kim CH, Muir-Hunter SW (2017). Risk factors associated with falls in older adults with dementia: a systematic review. Physiother Can.

[8] Yeung SSY, Reijnierse EM, Pham VK, Trappenburg MC, Lim WK, Meskers CGM, Maier AB (2019). Sarcopenia and its association with falls and fractures in older adults: a systematic review and meta-analysis. J Cachexia Sarcopenia Muscle.

[9] Brassington GS, King AC, Bliwise DL (2000). Sleep problems as a risk factor for falls in a sample of community-dwelling adults aged 64-99 years. J Am Geriatr Soc.

[10] Seppala LJ, Wermelink AMAT, De VM, Ploegmakers KJ, de Glind EMM V, Daams JG, Van der Velde N (2018). Fall-Risk-Increasing Drugs: A Systematic Review and Meta-Analysis: II. Psychotropics. J Am Med Dir Assoc.

[11] De VM, Seppala LJ, Daams JG, de Glind EMM V, Masud T, Van der Velde N (2018). Fall-Risk-Increasing Drugs: A Systematic Review and Meta-Analysis: I. Cardiovascular Drugs. J Am Med Dir Assoc.

[12] Seppala LJ, van de Glind EMM, Daams JG, Ploegmakers KJ, De VM, AMAT W, Van der Velde N (2018). Fall-Risk-Increasing Drugs: A Systematic Review and Meta-analysis: III. Others. J Am Med Dir Assoc.

[13] Friedman SM, Munoz B, West SK, Rubin GS, Fried LP (2002). Falls and fear of falling: which comes first? A longitudinal prediction model suggests strategies for primary and secondary prevention. J Am Geriatr Soc.

[14] Toebes MJP, Hoozemans MJM, Furrer R, Dekker J, van Dieën JH (2012). Local dynamic stability and variability of gait are associated with fall history in elderly subjects. Gait Posture.

[15] Chamberlin ME, Fulwider BD, Sanders SL, Medeiros JM (2005). Does fear of falling influence spatial and temporal gait parameters in elderly persons beyond changes associated with normal aging?. J Gerontol A Biol Sci Med Sci.

[16] Ayoubi F, Launay CP, Kabeshova A, Fantino B, Annweiler C, Beauchet O (2014). The influence of fear of falling on gait variability: results from a large elderly population-based cross-sectional study. J Neuroeng Rehabil.

[17] Reelick MF, van Iersel MB, Kessels RPC, Rikkert MGMO (2009). The influence of fear of falling on gait and balance in older people. Age Ageing.

[18] Maki BE (2015). Gait changes in older adults: predictors of falls or indicators of fear. J Am Geriatr Soc.

[19] Toebes MJP, Hoozemans MJM, Furrer R, Dekker J, van Dieën JH (2015). Associations between measures of gait stability, leg strength and fear of falling. Gait Posture..

[20] Thaler-Kall K, Peters A, Thorand B, Grill E, Autenrieth CS, Horsch A, Meisinger C (2015). Description of spatio-temporal gait parameters in elderly people and their association with history of falls: results of the population-based cross-sectional KORA-age study. BMC Geriatr.

[21] Niederer D, Beck V, Vogt L, Thiel C, Maulbecker-Armstrong C, Banzer W (2013). Bewegungsparcours, Sturzrisiko und gesundheitsbezogene Lebensqualität : Effekte einer 3-monatigen Bewegungsintervention. [activity trails, risk of falling, and health-related quality of life. Effects of a 12-week guided intervention]. Z Gerontol Geriatr.

[22] Fleckenstein J, Matura S, Engeroff T, Füzéki E, Tesky VA, Pilatus U, Hattingen E, Deichmann R, Vogt L, Banzer W, Pantel J (2015). SMART: physical activity and cerebral metabolism in older people: study protocol for a randomised controlled trial. Trials..

[23] Cordes T, Bischoff LL, Schoene D, Schott N, Voelcker-Rehage C, Meixner C, Appelles LM, Bebenek M, Berwinkel A, Hildebrand C, Jöllenbeck T, Johnen B, Kemmler W, Klotzbier T, Korbus H, Rudisch J, Vogt L, Weigelt M, Wittelsberger R, Zwingmann K, Wollesen B (2019). A multicomponent exercise intervention to improve physical functioning, cognition and psychosocial well-being in elderly nursing home residents: a study protocol of a randomized controlled trial in the PROCARE (prevention and occupational health in long-term care) project. BMC Geriatr.

[24] Matura S, Fleckenstein J, Deichmann R, Engeroff T, Füzéki E, Hattingen E, Hellweg R, Lienerth B, Pilatus U, Schwarz S, Tesky VA, Vogt L, Banzer W, Pantel J (2017). Effects of aerobic exercise on brain metabolism and grey matter volume in older adults: results of the randomised controlled SMART trial. Transl Psychiatry.

[25] Bikowski RM, Ripsin CM, Lorraine VL (2001). Physician-patient congruence regarding medication regimens. J Am Geriatr Soc.

[26] Mackenzie L, Byles J, D'Este C (2006). Validation of self-reported fall events in intervention studies. Clin Rehabil.

[27] Yardley L, Beyer N, Hauer K, Kempen G, Piot-Ziegler C, Todd C (2005). Development and initial validation of the falls efficacy scale-international (FES-I). Age Ageing.

[28] Kempen GIJM, Yardley L, JCM VH, GAR Z, Beyer N, Hauer K, Todd C (2008). The Short FES-I: a shortened version of the falls efficacy scale-international to assess fear of falling. Age Ageing.

[29] Kempen GIJM, Todd CJ, JCM VH, GAR Z, Beyer N, Freiberger E (2007). Cross-cultural validation of the Falls Efficacy Scale International (FES-I) in older people: results from Germany, the Netherlands and the UK were satisfactory. Disabil Rehabil.

[30] Osoba MY, Rao AK, Agrawal SK, Lalwani AK (2019). Balance and gait in the elderly: a contemporary review. Laryngoscope Investig Otolaryngol.

[31] Studenski S, Perera S, Patel K, Rosano C, Faulkner K, Inzitari M, Brach J, Chandler J, Cawthon P, Connor EB, Nevitt M, Visser M, Kritchevsky S, Badinelli S, Harris T, Newman AB, Cauley J, Ferrucci L, Guralnik J (2011). Gait speed and survival in older adults. JAMA..

[32] Kwon M-S, Kwon Y-R, Park Y-S, Kim J-W (2018). Comparison of gait patterns in elderly fallers and non-fallers. Technol Health Care.

[33] Fernandez NB, Hars M, Trombetti A, Vuilleumier P (2019). Age-related changes in attention control and their relationship with gait performance in older adults with high risk of falls. Neuroimage..

[34] Zhao R, Bu W, Chen X (2019). The efficacy and safety of exercise for prevention of fall-related injuries in older people with different health conditions, and differing intervention protocols: a meta-analysis of randomized controlled trials. BMC Geriatr.

[35] Thanapluetiwong S, Chewcharat A, Takkavatakarn K, Praditpornsilpa K, Eiam-Ong S, Susantitaphong P (2020). Vitamin D supplement on prevention of fall and fracture: A Meta-analysis of Randomized Controlled Trials. Medicine (Baltimore).

[36] Okubo Y, Schoene D, Lord SR (2017). Step training improves reaction time, gait and balance and reduces falls in older people: a systematic review and meta-analysis. Br J Sports Med.

[37] Mitchell RJ, Lord SR, Harvey LA, Close JCT (2014). Associations between obesity and overweight and fall risk, health status and quality of life in older people. Aust N Z J Public Health.

[38] Pirker W, Katzenschlager R (2017). Gait disorders in adults and the elderly : a clinical guide. Wien Klin Wochenschr.

